# Morphine-Induced Preconditioning: Involvement of Protein Kinase A and Mitochondrial Permeability Transition Pore

**DOI:** 10.1371/journal.pone.0151025

**Published:** 2016-03-11

**Authors:** Marianne Dorsch, Friederike Behmenburg, Miriam Raible, Dominic Blase, Hilbert Grievink, Markus W. Hollmann, André Heinen, Ragnar Huhn

**Affiliations:** 1 Department of Anesthesiology, University Hospital Duesseldorf, Moorenstr. 5, 40225, Duesseldorf, Germany; 2 Department of Anesthesiology, Laboratory of Experimental Intensive Care and Anesthesiology (L.E.I.C.A.), Academic Medical Center (AMC), University of Amsterdam, Meibergdreef 9, 1100 DD, Amsterdam, The Netherlands; 3 Institute of Cardiovascular Physiology, Heinrich-Heine-University, Universitaetsstr. 1, 40225, Duesseldorf, Germany; 4 Department of Anesthesiology and Critical Care Medicine, Hadassah University Hospital, Jerusalem, Israel; 5 Department of Biochemistry and Molecular Biology, The Hebrew University of Jerusalem, Ein Kerem Campus, Jerusalem, Israel; Indiana University School of Medicine, UNITED STATES

## Abstract

**Background:**

Morphine induces myocardial preconditioning (M-PC) via activation of mitochondrial large conductance Ca^2+^-sensitive potassium (mK_Ca_) channels. An upstream regulator of mK_Ca_ channels is protein kinase A (PKA). Furthermore, mK_Ca_ channel activation regulates mitochondrial bioenergetics and thereby prevents opening of the mitochondrial permeability transition pore (mPTP). Here, we investigated in the rat heart *in vivo* whether 1) M-PC is mediated by activation of PKA, and 2) pharmacological opening of the mPTP abolishes the cardioprotective effect of M-PC and 3) M-PC is critically dependent on STAT3 activation, which is located upstream of mPTP within the signalling pathway.

**Methods:**

Male Wistar rats were randomised to six groups (each n = 6). All animals underwent 25 minutes of regional myocardial ischemia and 120 minutes of reperfusion. Control animals (Con) were not further treated. Morphine preconditioning was initiated by intravenous administration of 0.3 mg/kg morphine (M-PC). The PKA blocker H-89 (10 μg/kg) was investigated with and without morphine (H-89+M-PC, H-89). We determined the effect of mPTP opening with atractyloside (5 mg/kg) with and without morphine (Atr+M-PC, Atr). Furthermore, the effect of morphine on PKA activity was tested in isolated adult rat cardiomyocytes. In further experiments in isolated hearts we tested the protective properties of morphine in the presence of STAT3 inhibition, and whether pharmacological prevention of the mPTP-opening by cyclosporine A (CsA) is cardioprotective in the presence of STAT3 inhibition.

**Results:**

Morphine reduced infarct size from 64±5% to 39±9% (P<0.05 vs. Con). H-89 completely blocked preconditioning by morphine (64±9%; P<0.05 vs. M-PC), but H-89 itself had not effect on infarct size (61±10%; P>0.05 vs. Con). Also, atractyloside abolished infarct size reduction of morphine completely (65±9%; P<0.05 vs. M-PC) but had no influence on infarct size itself (64±5%; P>0.05 vs. Con). In isolated hearts STAT3 inhibitor Stattic completely abolished morphine-induced preconditioning. Administration of Stattic and mPTP inhibitor cyclosporine A reduced infarct size to 31±6% (Stat+CsA, P<0.05 vs. Con). Cyclosporine A alone reduced infarct size to 26±7% (CsA P<0.05 vs. Con). In cardiomyocytes, PKA activity was increased by morphine.

**Conclusion:**

Our data suggest that morphine-induced cardioprotection is mediated by STAT3-activation and inhibition of mPTP, with STA3 located upstream of mPTP. There is some evidence that protein kinase A is involved within the signalling pathway.

## Introduction

Ischemic preconditioning is a phenomenon where short sublethal epidsodes of myocardial ischemia protect the heart against the consequences of a longer myocardial ischemic period and thus against ischemia/reperfusion injury [1]. Short cycles of ischemia and reperfusion at the end of a longer ischemia and at the beginning of reperfusion can also protect the heart against ischemia/reperfusion injury; a phenomenon called ischemic postconditioning [2]. Beside ischemic stimuli the cardioprotective effect of pre- and postconditioning can be mimicked by volatile anesthetics [3,4] but also with morphine [5]. The cardioprotective effect of pre- and postconditioning involve amongst others mitochondrial potassium channels and the mitochondrial permeability transition pore “mPTP” [6].

Recently, we could demonstrate that the cardioprotective effect of morphine was blocked by the mitochondrial calcium-sensitive potassium channel (mK_Ca_) inhibitor paxilline [7,8], indicating a crucial role of mK_Ca_ channels in morphine-induced pre- and postconditioning. Furthermore, Cao et al. [9] could show that the cardioprotective effect of mK_Ca_ channel activation was abolished by opening of the mPTP, suggesting that the mK_Ca_ channel is an upstream regulator of the mPTP. The blockade of morphine-induced cardioprotection by paxilline leads to the question, how mK_Ca_ channel activation is regulated. A possible regulator of mK_Ca_ channels is protein kinase A (PKA, cAMP dependent protein kinase) [10,11], mK_Ca_ channels could be activated by PKA and PKA in turn is regulated by cellular cAMP levels.

We hypothesize that the cardioprotective effect of morphine is mediated by activation of PKA and regulation of mPTP.

## Methods

The study was performed in accordance with the guidelines laid out in the Guide for the Care and Use of Laboratory Animals, which is available from the National Academy of Science and the regulations of the German Animal Protection Law and was approved by the District Government of Duesseldorf, Germany (8.87–51.04.20.09.388).

### Surgical preparation

Male Wistar rats had free access to water and standard rat food at all times prior to experiments. The animal preparation was performed as described previously [12,13]. Animals (296±30 g) were instrumented with a coronary occluder around a left anterior descending artery. Pentobarbital-anesthetized animals were endotracheal intubated with a plastic cannula (outer diameter 2.2 mm). After a median incision was performed on cervical level a 20 gauge cannula was inserted into the right internal carotid artery and advanced into the aorta in order to measure the aortic pressure. Aortic pressure was digitized using an analogue to digital converter (PowerLab/8SP, ADInstruments Pty Ltd, Castle Hill, Australia) at a sampling rate of 500 Hz and were continuously recorded on a personal computer using Chart for Windows v5.0 (ADInstruments Pty Ltd, Castle Hill, Australia). All animals underwent 25 min of coronary artery occlusion followed by 2 h reperfusion. At the end of reperfusion the hearts were excised and infarct sizes were determined using a previously described method [14,15]. Briefly, the heart was excised with the occluding suture left in place and then mounted on a modified Langendorff apparatus for perfusion with ice cold normal saline via the aortic root at a perfusion pressure of 80 cm H_2_O in order to wash out intravascular blood. After 5 min of perfusion, the coronary artery was re-occluded and the remainder of the myocardium was perfused through the aortic root with 0.2% Evans blue in normal saline for 10 min. Intravascular Evans blue was then washed out by perfusion with normal saline for 10 min. This treatment identified the area at risk as unstained. The heart was then cut into 2 mm thick transverse slices. The slices were stained with 0.75% triphenyltetrazolium chloride solution for 10 min at 37°C, and fixed in 4% formalin solution for 24 h at room temperature. The area at risk and the infarct size were determined using planimetry and corrected for dry weight in each slice by using SigmaScan Pro5^®^ (SPSS Science Software, Chicago, IL, USA).

### Experimental protocol

**Fig 1:**
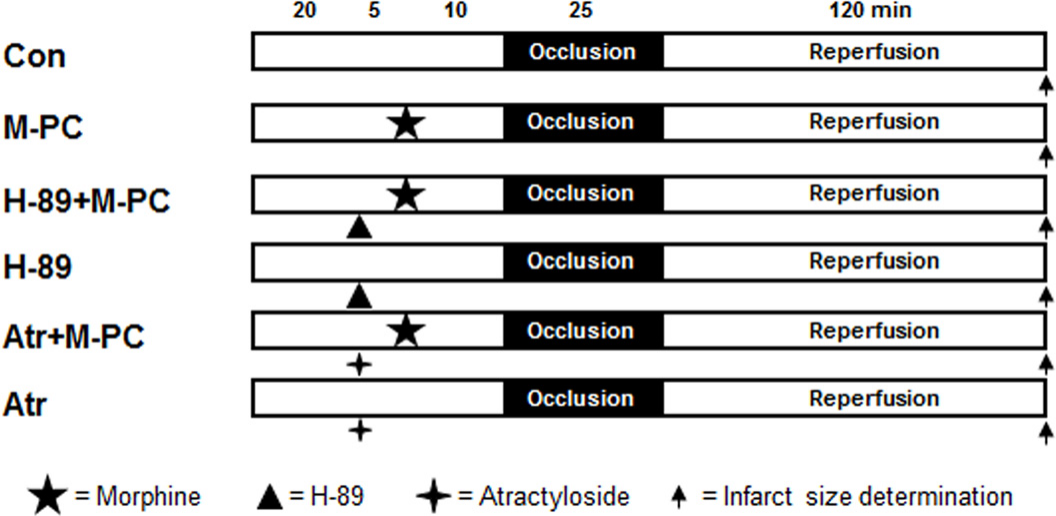
Experimental protocol for *in vivo* infarct size measurement. Con = control, M-PC = morphine preconditioning, H-89 = protein kinase A inhibitor, Atr = atractyloside (mPTP opener).

Rats for infarct size experiments were randomly allocated into six groups (see Fig 1). Morphine (0.3 mg/kg) [16] was administered intravenously 10 minutes before ischemia. The PKA inhibitor H-89 (10 μg/kg) [17] and the mPTP opener atractyloside (5 mg/kg) [18] were given 15 minutes before the onset of ischemia. At the end of the experiments hearts were excised and infarct sizes were determined using a previously described method [14].

### Isolated heart experiments

Surgical preparation was performed as described previously. In brief, rats were anesthetized by intraperitoneal injection of pentobarbital (90 mg/kg). After thoracotomy hearts were excised and mounted on a Langendorff system. Perfusion of the hearts was performed at constant pressure (80 mmHg) with a Krebs-Henseleit solution, containing (in mM): 116 NaCl, 4.7 KCl, 1.1 MgSO4, 1.17 KH2PO4, 24.9 NaHCO3, 2.52 CaCl2, 8.3 glucose, and 2.2 pyruvate at 37°C. A fluid filled balloon was inserted into the left ventricle and end-diastolic pressure was set at 1–4 mmHg. All hearts underwent an equilibration period of 20 minutes. Thereafter, heart rate, the rate pressure product (RPP, calculated as heart rate x (maximal left ventricular pressure–minimal left ventricular pressure)), left ventricular end-diastolic pressure (LVEDP), and coronary flow were measured continuously and digitized using an analogue to digital converter (PowerLab/8SP, ADInstruments Pty Ltd, Castle Hill, Australia) at a sampling rate of 500 Hz. The data were continuously recorded on a personal computer using Chart for Windows v5.0 (ADInstruments Pty Ltd, Castle Hill, Australia). Maximal contracture and time to maximal contracture were detected by checking the course of contracture development during index ischemia and selecting the time point when contracture reached its highest level in each experiment. Arrhythmic intervals were not used for data analysis.

### Experimental protocol

**Fig 2:**
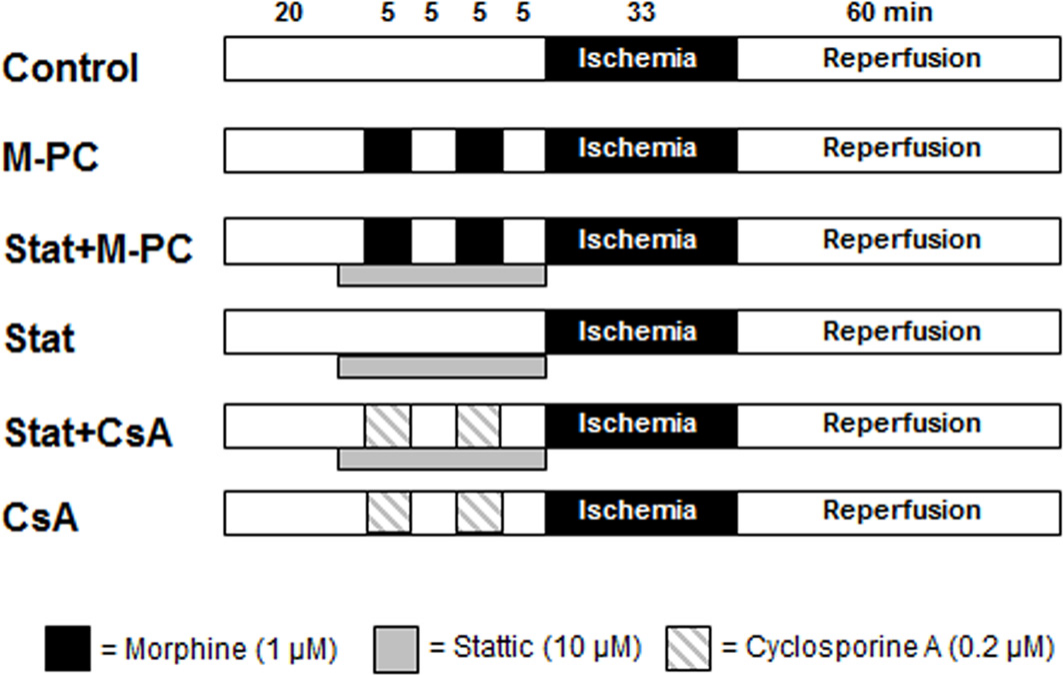
Experimental protocol for *in vitro* infarct size measurement. Con = control, M-PC = morphine preconditioning, Stat = STAT3 inhibitor stattic, CsA = atractyloside (mPTP blocker).

Fig 2 summarizes the experimental design. Hearts were randomly assigned to one of six experimental groups. In group 1 (Control) hearts were kept under baseline conditions for 35 minutes before they underwent 33 minutes of ischemia followed by 60 minutes of reperfusion. Morphine (1 μM) and mPTP inhibitor cyclosporine A (0.2 μM) [19] were administered twice for 5 minutes starting 20 and 10 minutes before onset of ischemia. STAT3 inhibitor Stattic (10 μM) [20] was administered over 23 minutes before performing global ischemia.

After 60 minutes of reperfusion, the hearts were cut into transverse slices, which were then stained with 0.75% triphenyltetrazoliumchloride (TTC) solution. The infarcted area was determined by planimetry using SigmaScan Pro 5^®^ computer software (SPSS Science Software, Chicago, IL).

### Cardiomyocytes

To investigate whether morphine regulates PKA activity, three additional experimental groups (Con, Morphine 1 μM, Morphine 10 μM, each n = 7) were determined. Cardiomyocytes were isolated from male Wistar rats. Hearts were perfused with oxygen-enriched medium (NaCl 110 mmol/l, KH_2_PO_4_ 1.2 mmol/l, KCl 2.5 mmol/l, MgSO_4_ 1.2 mmol/l, HEPES 25 mmol/l, glucose 10 mmol/l; perfusion flow 10 ml/min). Subsequently, recirculating perfusion was continued for 25 minutes (5 ml/min) with added ~0.06 w/v% collagenase and 25 μM CaCl_2_. Afterwards, the ventricular mass was minced into small pieces and incubated at 37°C for 5 minutes in oxygen-enriched medium. To isolate intact cardiomyocytes from large cellular detritus, the cell suspension was filtrated through a nylon-mesh. The cell suspension was centrifuged 3 times for 2 minutes at 20 xg whilst gradually increasing CaCl_2_ concentrations (0.2 mmol/l, 0.4 mmol/l and 1.0 mmol/l). The resulting pellet was resuspended in serum free M199 (Biochrom AG, Berlin, Germany; supplemented with pen/strep 2%, HEPES 15 mmol/l, L-carnitine 2 mmol/l, creatine 5 mmol/l, taurine 5 mmol/l and 10 μmol/l cytosin-arabinofuranosid) and plated out on pre-coated (4 v/v% fetal calf serum) culture dishes. After 1.5–2 hours of incubation, cardiomyocytes adhered and non-viable cells were washed off leading to cultures of >90% rod shaped cells. The percentage of vital cardiomyocytes was quantified with trypan blue staining.

### Western blot analysis

As a marker for PKA activation, phosphorylation of phospholamban, a protein that is phosphorylated by PKA, was determined. Western blot analysis was performed as described previously [15].

### Statistical analysis

The sample size was calculated using GraphPad StatMate^TM^ Version 1.01 (GraphPad Software, San Diego, CA, USA). Sample size analysis revealed a group size of n = 6 was necessary to detect a difference in infarct size of 14% with a power of 80% and α<0.05. Data from infarct size analysis are expressed as mean and standard deviation (SD), data from western blot analysis as median with 25th and 75th percentiles. Statistical analysis (SPSS Science Software, version 17.0) of the hemodynamic variables was performed by using a two-way analysis of variance followed by Tukey test. Infarct sizes were analysed by a one-way analysis of variance followed by Tukey test. Statistical analysis of the western blot data was performed by the Kruskal–Wallis H-test followed by the Student–Newman–Keuls post hoc test. Changes within and among groups were considered statistically significant if P<0.05.

## Results

### Infarct size measurement

**Fig 3:**
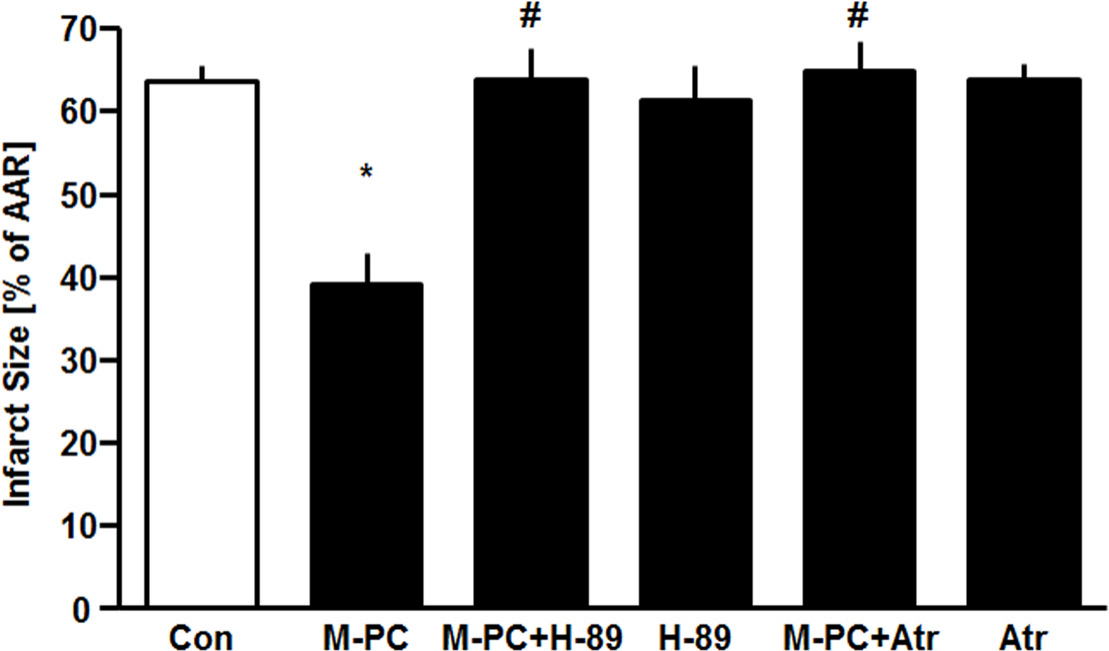
Infarct size measurement. Histogram shows the infarct size (percent of area at risk) of controls (Con), morphine preconditioning (M-PC), H-89 combined with morphine preconditioning (H-89+M-PC), H-89 alone (H-89), atractyloside combined with morphine preconditioning (Atr+M-PC) and atractyloside alone (Atr). Data are presented as mean±SD, *P<0.05 vs. Con, ^#^P<0.05 vs. M-PC.

Administration of morphine *in vivo* reduced infarct size from 64±5% in the control group to 39±9% (P<0.05, see Fig 3). In combination with PKA-Inhibitor H-89 the effect of morphine was completely abolished (H-89+M-PC: 63±10%, P<0.05 vs. M-PC). Single administration of H-89 did not show any reduction of the infarct size compared to the control group (H-89: 61±10%, ns vs. Con).

The cardioprotective effect of morphine was completely abolished by atractyloside (Atr+M-PC: 65±9%, P<0.05 vs. M-PC) suggesting a critical role for prevention of mPTP opening in M-PC. Atractyloside alone had no effect on infarct size (Atr: 64±5%, ns vs. Con).

**Fig 4:**
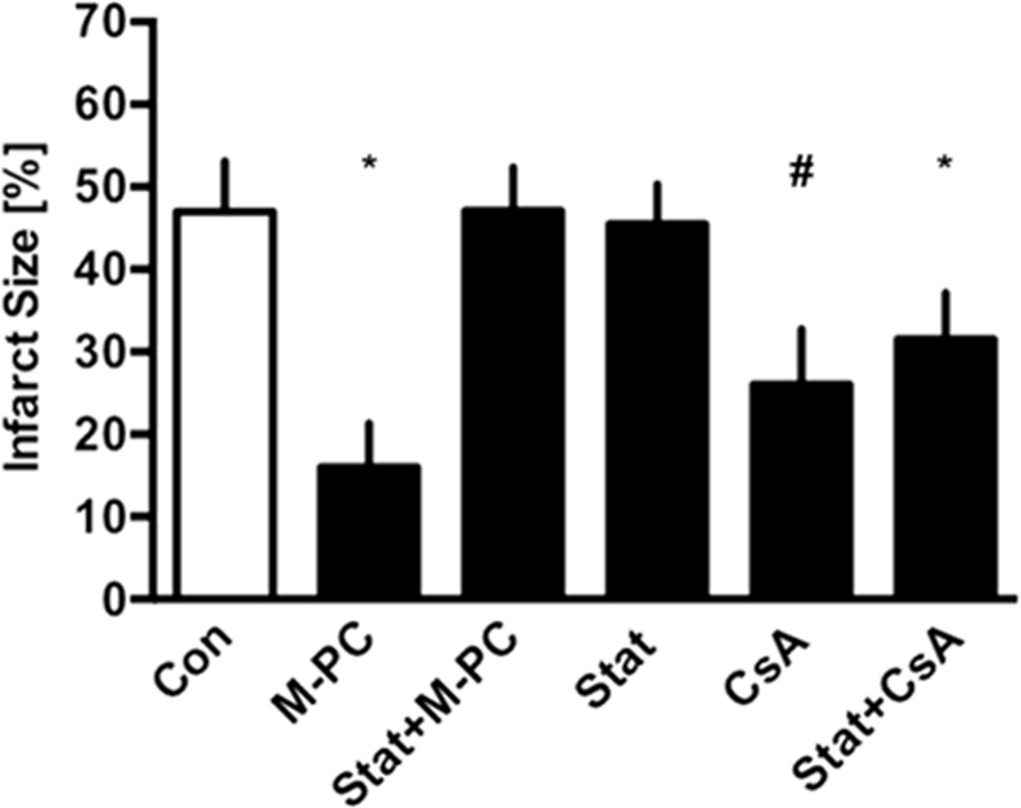
Infarct size measurement. Histogram shows the infarct size of controls (Con), morphine preconditioning (M-PC), STAT3-inhibitor Stattic and morphine preconditioning (Stat+M-PC), Stattic alone (Stat), Stattic combined with mPTP-blocker cyclosporine A (Stattic and CsA) and cyclosporine A alone (CsA). Data are presented as mean±SD, *P<0.05 vs. Con, ^#^P<0.05 vs. M-PC.

In Langendorff experiments administration of morphine reduced infarct size from 47±6% in the control group to 16±5% (see Fig 4). In combination with STAT3 inhibitor Stattic the effect was completely abolished (Stat+M-PC, 47±5% P<0.05 vs. Con). Stattic alone had no effect on infarct size (Stat 45±5%, P<0.05 vs. M-PC). Administration of Stattic and mPTP inhibitor cyclosporine A reduced infarct size to 31±6% (Stat+CsA, P<0.05 vs. Con). Cyclosporine A alone reduced infarct size to 26±7% (CsA P<0.05 vs. Con).

### Hemodynamic variables

As shown in Table 1, no significant differences in heart rate and aortic pressure were observed among the *in vivo* experimental groups during baseline, ischemia or reperfusion. After 120 minutes of reperfusion, heart rate was significantly lower in the H-89+M-PC and in the Atr group (P<0.05 vs. baseline) compared to baseline. Mean aortic pressure was significantly decreased at the end of reperfusion in the Con and Atr group (P<0.05 vs. baseline). As summarized in Table 2 at none of the different time points during Langendorff experiments was there a significant difference in the heart rate, RPP, LVEDP, and coronary blood flow among the different experimental groups. Over the duration of the experiments heart rate remained stable in all groups, whereas LVEDP was increased during the reperfusion period (all P<0.05 versus baseline). In contrast, coronary flow decreased during the reperfusion period (P<0.05 versus baseline).

**Table 1:** Hemodynamic variables.

	Baseline	Ischemia	Reperfusion	
		15	30	120
*Heart Rate (bpm)*				
Con	357±32	345±22	323±47	290±46
M-PC	367±24	353±24	319±68	297±41
H-89+M-PC	387±27	361±29	326±52	302±73*
H-89	380±72	352±52	318±40	292±25
Atr+M-PC	378±86	342±62	312±41	302±32
Atr	391±36	392±26	332±25	293±23*
*Mean AOP (mmHg)*						
Con	101±21	94±17	76±17	62± 7*
M-PC	102±12	94± 9	81±18	66±24
H-89+M-PC	108±18	95±25	82±31	80±43
H-89	108±39	91±39	76±25	64± 7
Atr+M-PC	109±45	97±36	84±30	69±23
Atr	113±23	102±35	82±27	65±17*

Data are mean±SD. Con = control; M-PC = morphine preconditioning; H-89+M-PC = PKA inhibitor H-89 prior to morphine preconditioning; H-89 = PKA inhibitor H-89 alone; Atr+M-PC = mPTP opener atractyloside prior to morphine preconditioning; Atr = atracyloside alone

*p<0.05 vs. Baseline.

**Table 2:** Hemodynamic variables.

	Baseline	Reperfusion		
		30	45	60
*Heart Rate (bpm)*				
Con	325 ± 41	274 ± 53	292 ± 28	267 ± 40
M-PC	334 ± 48	297 ± 33	282 ± 44	267 ± 43
Stat+M-PC	303 ± 12	272 ± 59	271 ± 37	250 ± 55
Stat	297 ± 27	259 ± 51	248 ± 60	247 ± 44
Stat+CsA	330 ± 26	270 ± 25	289 ± 33	292 ± 38
CsA	308 ± 43	241 ± 39	218 ± 52	253 ± 61
*LVEDP (mmHg)*				
Con	3 ± 2	68 ± 10*	62 ± 9*	60 ± 8*
M-PC	4 ± 1	71 ± 7*	65 ± 5*	63 ± 5*
Stat+M-PC	3 ± 2	69 ± 11*	66 ± 9*	63 ± 8*
Stat	4 ± 2	70 ± 11*	66 ± 10*	64 ± 9*
Stat+CsA	3 ± 2	60 ± 10*	55 ± 11*	52 ± 9*
CsA	5 ± 3	72 ± 7*	67 ± 6*	63 ± 5*
CF (ml*min^*-1*^*)*				
Con	15 ± 2	10 ± 2*	10 ± 2*	10 ± 2*
M-PC	14 ± 2	10 ± 2*	10 ± 3*	10 ± 3*
Stat+M-PC	13 ± 3	7 ± 2*	7 ± 2*	7 ± 2*
Stat	13 ± 3	7 ± 2*	7 ± 2*^§^	6 ± 2*^§^
Stat+CsA	14 ± 2	8 ± 2*	7 ± 2*	7 ± 2*
CsA	13 ± 2	8 ± 1*	8 ± 1*	7 ± 1*

Data are mean±SD. Con = control; M-PC = morphine preconditioning; Stat = stattic; CsA = cyclosporine A.

*P<0.05 vs. Baseline

^§^P<0.05 vs. Con.

### Animal characteristics

There were no differences in body weight or heart weight, respectively, between the different experimental groups (see Table 3).

**Table 3:** Weights and ischemic contracture.

	n	Heart weight wet (g)	Heart weight dry (g)	Maximal ischemic contracture (mmHg)	Time of maximal ischemic contracture (min)
Control	7	1.56 ± 0.10	0.14 ± 0.01	51 ± 14	15 ± 2
M-PC	7	1.57 ± 0.11	0.15 ± 0.01	59 ± 16	15 ± 1
Stat+M-PC	7	1.51 ± 0.06	0.14 ± 0.02	56 ± 6	13 ± 1
Stat	8	1.51 ± 0.18	0.15 ± 0.01	59 ± 6	15 ± 2
Stat+CsA	7	1.56 ± 0.10	0.15 ± 0.01	46 ± 10	14 ± 2
CsA	7	1.65 ± 0.04	0.14 ± 0.01	47 ± 9	15 ± 1

Data are mean±SD. M-PC = morphine preconditioning; Stat = stattic; CsA = cyclosporine A.

### Western blot analysis

**Fig 5:**
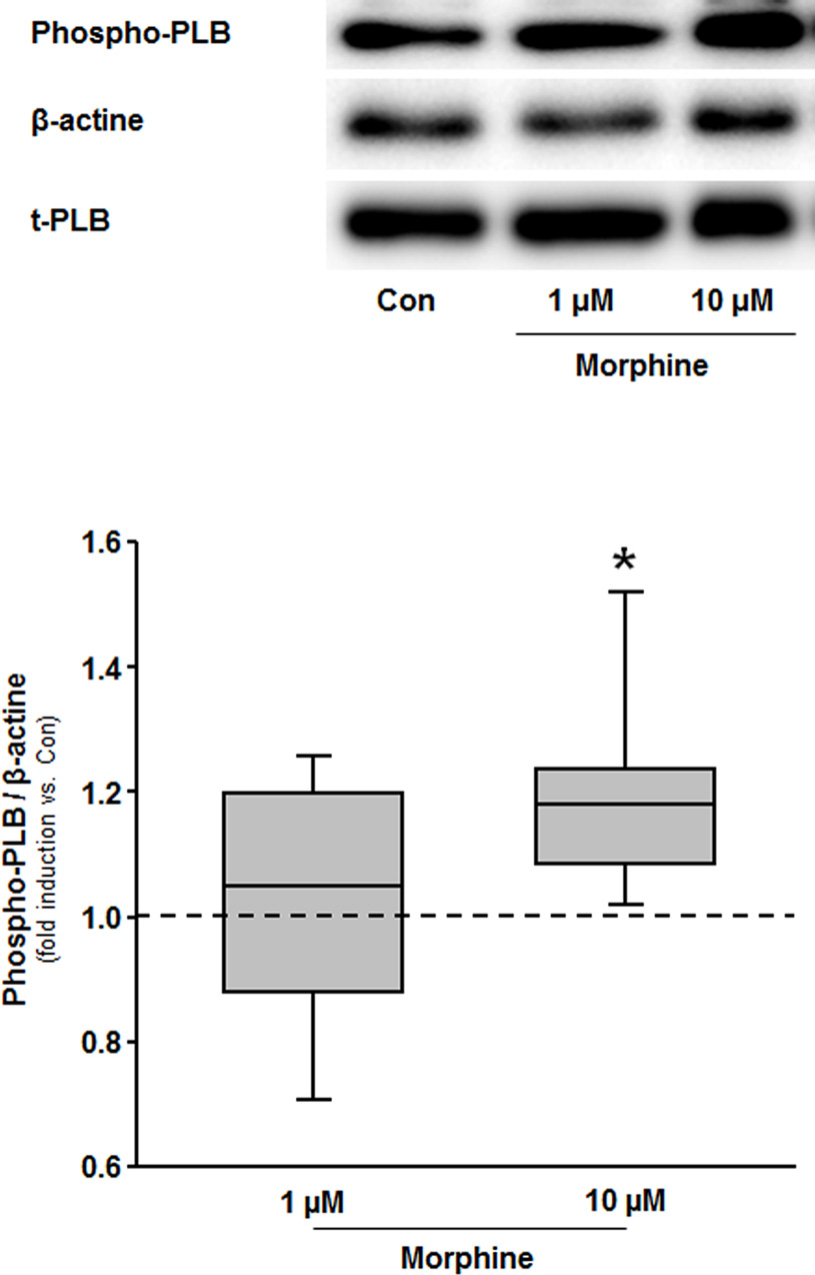
Western Blot analysis. Summarized data presenting ratio of phosphorylated phospholamban (phospho-PLB) to beta-actine from control and morphine treated hearts are shown. The boxes represent the lower (25th) and upper (75th) quartiles, the horizontal line represents the mean, the whiskers extend to 95% confidence intervals for the mean, *P<0.05 vs. Con.

Fig 5 illustrates that morphine in a concentration of 10 μM significantly increased phospholamban phosphorylation (1.21(0.19) vs. Con, P<0.05). In contrast, 1 μM morphine had no effect on phospholamban phosphorylation (1.03(0.21) ns vs. Con).

## Discussion

The main findings of our study suggest that morphine-induced preconditioning is mediated by activation of STAT3 which is located upstream of mPTP. Furthermore, there is some evidence that protein kinase A is involved within the signalling pathway.

Because morphine can protect the heart by pre- and postconditioning [7,8] it is suitable agent for patients undergoing organ ischemia (e.g. heart surgery, vascular surgery, organ transplantation) or for patients who were recently exposed to regional ischemia (e.g. stroke, angina pectoris, myocardial infarction). Therefore, it is of particular interest to elucidate the underlying mechanisms of morphine-induced cardioprotection.

Previously, we could demonstrate that morphine-induced preconditioning is mediated by activation of mK_Ca_ channels [7]. The mK_Ca_ channel inhibitor paxilline completely abrogated the cardioprotective effect of morphine [7]. Cao et al. could show that the cardioprotective effect of mK_Ca_ channel activation is abolished by opening of the mPTP [9]. The authors concluded that the mK_Ca_ channel is an upstream regulator of the mPTP. This conclusion was confirmed by the fact that inhibition of the mK_Ca_ channel with paxilline and inhibition of the mPTP with cyclosporine A at the same time was still protective [9]. Together with our own data showing that morphine preconditioning is mediated by activation of mK_Ca_ channels [7] it is possible that activation of the mK_Ca_ channel is an intermediate step in the signal transduction cascade of morphine preconditioning. A possible upstream regulator of mK_Ca_ channels is protein kinase A [10,11]. For desflurane preconditioning protein kinase A is located upstream of mK_Ca_ channels [21]. The activity of protein kinase A depends on the cellular level of cAMP. Morphine can lead to an increase in cellular cAMP levels [22] but the underlying mechanism is unknown. However, it is conceivable that the increase in cAMP levels depends either on an elevated synthesis of cAMP by stimulation of adenylate cyclase or a less prominent decrease in cAMP by inhibition of phosphodiesterase. The first aim of the present study was to investigate the signal pathway of morphine preconditioning consisting of protein kinase A activation via mK_Ca_ channel and inhibition of mPTP opening. We can exclude that the protective effect of morphine is caused by differences in mechanical heart function as we determined no differences in left ventricular function or coronary flow at baseline or during drug administration among treatment groups. Additionally we do not see significant differences in ischemic contracture or heart weights among the groups.

Our finding that the protein kinase A inhibitor H-89 completely abolished the cardioprotective effect of morphine suggest that morphine-induced preconditioning is critically dependent on activation of protein kinase A. However, this conclusion is challenged by investigations demonstrating non-specific actions of H-89 [23].

Interestingly, the existence of a possible mechanistic link between morphine and PKA might be supported by our finding that morphine increases phospholamban phosphorylation. It is important to note that this effect was seen using a high morphine concentration of 10 μM. This concentration is usually not reached in blood plasma after morphine bolus injection and therefore has to be considered as "supra-pharmacological" concentration. In conclusion, our findings 1) that morphine-induced cardioprotection is abrogated by PKA inhibitor H-89, and 2) morphine is capable to activate PKA in cardiomyocytes at least at 10 μM might indicate that PKA activation is involved in morphine-induced preconditioning.

In addition, it has been demonstrated that activation of protein kinase A can regulate the mK_Ca_ channels [10,11]. Redel et al demonstrated using a desflurane-induced preconditioning model that protein kinase A is located upstream of the mK_Ca_ channel [21].

**Fig 6:**
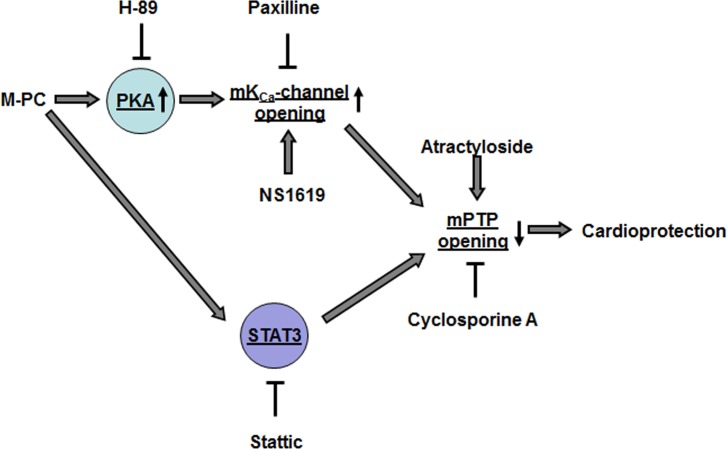
Schematic model of the proposed cardioprotective pathway of morphine preconditioning. M-PC = morphine preconditioning, PKA = protein kinase A, H-89 = protein kinase A inhibitor, Paxilline = mKCa-channel inhibitor, NS1619 = mKCa-channel activator, mPTP = mitochondrial permeability transition pore, Stattic = STAT3 inhibitor

Mitochondrial mechanisms are involved in myocardial pre- and postconditioning. Activation of mitochondrial potassium channels might lead to an inhibition of mPTP opening [24,25]. Hausenloy et al. reported that inhibition of mPTP is involved in cardioprotection by ischemic preconditioning [26]. Potassium influx into the mitochondrial matrix via activated potassium channels was suggested to change mitochondrial function. This change leads to the release of oxygen radicals, probably caused by a loss of electrons from the mitochondrial electron transport chain. These oxygen radicals are crucially involved in activating different kinases, which act as trigger and/or mediator of cardioprotection [27,28]. Mitochondria might play a role as an endeffector of cardioprotection. A possible candidate is the mPTP [26,29]. As mentioned above the mPTP is located downstream of mK_Ca_ channels [9]. Based on the results from this study together with data from a recent publication of our group showing that morphine preconditioning is abolished by inhibition of mK_Ca_ channels [30], we suggest the following signal transduction pathway for morphine preconditioning (see Fig 6): Morphine enhances protein kinase A activity, which in turn leads to activation of mK_Ca_ channels with the consequence of prevention of mPTP opening during myocardial ischemia and reperfusion.

However, despite the significance of these findings, our results represent only a small proportion of mechanisms underlying morphine-induced cardioprotection. Other signalling pathways have been described for morphine-induced preconditioning including a role for PI3K/Akt signalling and/or JAK/STAT3 activation. To integrate our results into some aspects of other signalling mechanisms described we conducted supplementary experiments in isolated hearts focusing on the role of STAT3. It has been demonstrated that STAT3 plays an essential role in morphine-induced cardioprotection [16]. Furthermore there is evidence that STAT3 might be regulated by activation of PKA [31] and is mechanistically linked to prevention of mPTP-opening [32].Therefore, we tested the protective properties of morphine in the presence of STAT3 inhibitior Stattic, and whether pharmacological prevention of the mPTP-opening by cyclosporine A (CsA) is cardioprotective during STAT3 inhibition.

Our data show that the protective effect of morphine is abrogated by the STAT3 inhibitor Stattic. This is in line with results from other groups showing a role for STAT3 in morphine-PC. In addition, our finding that CsA offers cardioprotection during STAT3 inhibition suggests that mPTP might be located downstream of STAT3 within the protective signalling pathway.

It remains unclear whether the cAMP/PKA/mK_Ca_ signalling pathway and the STAT3 pathway are redundant signalling pathways that only converge at the mitochondrial level (mPTP) to initiate protection or whether these pathways directly interact, e.g. by PKA-STAT3 interaction. These specific questions have to be addressed in future studies.

Fig 6 shows a schematic representation of the signalling pathways of morphine-induced preconditioning that has been investigated within this study. It has to be mentioned that several other signalling pathways have been described to be involved in morphine-induced cardioprotection that are not included in this simplified model. Morphine initiates cardioprotection by activation of the PKA/mK_Ca_/mPTP pathway. Furthermore, morphine-induced cardioprotection is dependent on STAT3 activation; however it remains unclear whether morphine activates STAT3 via PKA dependent or independent mechanisms. Furthermore, within the signalling pathway, STAT3 is located upstream of mPTP.
